# Mild mitochondrial impairment activates overlapping longevity pathways converging on the flavin-containing monooxygenase FMO-2

**DOI:** 10.3389/fragi.2026.1808540

**Published:** 2026-03-25

**Authors:** Jeremy M. Van Raamsdonk

**Affiliations:** 1 Department of Neurology and Neurosurgery, McGill University, Montreal, QC, Canada; 2 Metabolic Disorders and Complications Program, Research Institute of the McGill University Health Centre, Montreal, QC, Canada; 3 Brain Repair and Integrative Neuroscience Program, Research Institute of the McGill University Health Centre, Montreal, QC, Canada; 4 Division of Experimental Medicine, Department of Medicine, McGill University, Montreal, QC, Canada

**Keywords:** aging, *C. elegans*, FMO-2, genetics, lifespan, mitochondria

## Abstract

A mild impairment of mitochondrial function activates the hypoxia inducible factor (HIF-1)-mediated hypoxia stress response pathway leading to a HIF-1-dependent increase in lifespan. Lifespan extension resulting from HIF-1 stabilization is dependent on activation of flavin-containing monooxygenase-2 (FMO-2). In this work, we explored the role of *fmo-2* in the long lifespan of genetic mitochondrial mutants in *C. elegans.* We found that *fmo-2,* but not other *fmo* genes, are specifically upregulated in the long-lived mitochondrial mutants *clk-1, isp-1* and *nuo-6.* Disruption of *fmo-2* through RNA interference or genetic mutation shortens the lifespan of these mitochondrial mutants indicating that *fmo-2* is required for lifespan extension in these worms. Moreover, signaling molecules that have been shown to be involved in upregulation of *fmo-2* are also required for the long life of *clk-1, isp-1* and *nuo-6* mutants including HLH-30, NHR-49 and MDT-15. Finally, we examined the effect of multiple lifespan-promoting pathways in *clk-1* mutants on the expression of *fmo-2.* We found that in all cases, genes required for *clk-1* longevity are also required for the upregulation of *fmo-2* in *clk-1* worms. These genes included DAF-16, PMK-1, SKN-1, CEH-23, AAK-2, HIF-1 and ELT-2. Combined, this work advances our understanding of the molecular mechanisms contributing to longevity in the long-lived mitochondrial mutants and identifies FMO-2 as a common downstream effector of multiple pathways that modulate longevity.

## 1 Introduction

Activation of the HIF-1 hypoxia pathway extends lifespan in *C. elegans* (Mehta et al., 2009; Zhang et al., 2009)*.* This can be achieved through either overexpression of *hif-1* or through disruption of *vhl-1*, which encodes the E3 ubiquitin ligase von Hippel-Lindau protein (VHL-1) that degrades HIF-1 under normoxia. Moreover, it has been shown that the HIF-1 hypoxia pathway is required for the extended longevity of multiple long-lived mitochondrial mutants including *clk-1, isp-1* and *nuo-6* (Lee et al., 2010; Wu et al., 2018).

To gain insight into the mechanisms by which the HIF-1 hypoxia pathway promotes longevity, an RNAi screen was performed to identify RNAi clones that inhibit the decrease in age-associated autofluorescence in long-lived *vhl-1* mutants (Leiser et al., 2015). They found that RNAi targeting the flavin-containing monooxygenase-2 gene *fmo-2* not only inhibits the reduction of autofluorescence in *vhl-1* worms but also decreases their extended longevity. Moreover, ubiquitously overexpressing *fmo-2* was found to be sufficient to increase lifespan.

The lifespan extension in *fmo-2* overexpression worms was found to be independent of the stress-responsive transcription factors HIF-1, DAF-16 and SKN-1. Disruption of *fmo-2* was found to also prevent lifespan extension resulting from dietary restriction but not *daf-2* RNAi or *isp-1* RNAi (Leiser et al., 2015).

In exploring mechanisms leading to *fmo-2* upregulation, it was found that the TFEB transcription factor HLH-30, which has been shown to regulate autophagy, is required for the full induction of *fmo-2* in response to hypoxia or dietary restriction (Leiser et al., 2015). HLH-30 is also required for lifespan extension resulting from disruption of *vhl-1* or dietary restriction (Lapierre et al., 2013; Leiser et al., 2015), thereby providing further links between *fmo-2* expression and longevity.

A subsequent study showed that RNAi knockdown of transaldolase (*tald-1)* increases lifespan and activates *fmo-2* in a HLH-30- and PMK-1-dependent manner (Bennett et al., 2017). Importantly, *fmo-2* is required for *tald-1* RNAi to increase lifespan as *tald-1* RNAi fails to extend longevity in *fmo-2* mutants. A further study into the mechanisms of *fmo-2* activation found that MDT-15, which encodes a subunit of the Mediator transcriptional coactivator complex, and NHR-49, which encodes a nuclear hormone receptor that acts with MDT-15 to regulate various aspects of metabolism, are both required for *fmo-2* activation (Goh et al., 2018). This study also demonstrated that the effect of NHR-49 on *fmo-2* activation is independent of HLH-30, and that SEK-1 and PMK-1 from the p38-mediated innate immune signaling pathway are also required for full *fmo-2* activation (Goh et al., 2018).

Finally, it was shown that exposing worms to heat-inactivated, exopolysaccharide-producing strains of lactobacilli results in increased expression of *fmo-2* and extended longevity (Jakovljevic et al., 2025). The increase in lifespan was found to be dependent on *fmo-2* as well as *hlh-30* and *nhr-49,* which are involved in *fmo-2* activation (Jakovljevic et al., 2025).

In this work, we further explore the role of *fmo-2* in longevity. We find that *fmo-2* is upregulated in a specific group of long-lived mutant strains, which includes the long-lived mitochondrial mutants *clk-1, isp-1* and *nuo-6.* We find that FMO-2 and proteins involved in regulating FMO-2 expression, including HLH-30, SEK-1, PMK-1, NHR-49 and MDT-15 are all required for the long-lifespan of the mitochondrial mutants. Moreover, genetic pathways that affect longevity in *clk-1* mutants tend to also affect the expression of *fmo-2.* Overall, this work shows that *fmo-2* is important in mediating the lifespan extension in long-lived mitochondrial mutants. While this work provides additional support for the positive relationship between *fmo-2* expression and longevity, it also provides counter examples in which long-lived mutants (*eat-2, osm-5*) have extended longevity despite decreased expression of *fmo-2.*

## 2 Materials and methods

### 2.1 Strains

Strains were maintained on NGM plates at 20 °C and fed OP50 *E. coli* bacteria. The following strains were used in this study: N2 (wild-type), *sod-2(ok1030), clk-1(qm30), isp-1(qm150), nuo-6(qm200), daf-2(e1370), glp-1(e2141), eat-2(ad1116), osm-5(p813), ife-2(ok306), fmo-2(ok2147), pmk-1(km25), ceh-23(ms23), aak-2(ok524)* and *hif-1(ia4).* All strains were outcrossed a minimum of three generations. Some of these strains were crossed together to generate the following double mutant strains: JVR453 *clk-1(qm30);fmo-2(ok2147),* JVR441 *nuo-6(qm200);fmo-2(ok2147),* JVR227 *clk-1(qm30);pmk-1(km25),* JVR099 *clk-1(qm30);ceh-23(ms23),* JVR038 *clk-1(qm30);aak-2(ok524),* and MQ1758 *clk-1(qm30);hif-1(ia4).*

### 2.2 RNA isolation

RNA was isolated from pre-fertile young adult worms as described previously (Machiela et al., 2016). Briefly, worms from an overnight limited lay were collected and washed three times in M9 buffer before being frozen in Trizol. RNA was isolated from three biological replicates collected on different days.

### 2.3 RNA sequencing

RNA sequencing was performed and processed previously (Senchuk et al., 2018; Wu et al., 2018; Soo et al., 2021; Harris-Gauthier et al., 2022). Raw sequencing data is available on NCBI GEO: GSE179825, GSE93724 and GSE110984. An online tool for looking up genes of interest in this panel of long-lived mutants may be found here: https://vanraamsdonk.shinyapps.io/mutant_comparison_viewer/.

### 2.4 Quantitative RT-PCR

For quantitative RT-PCR (qPCR), RNA was converted to cDNA using a High-Capacity cDNA Reverse Transcription Kit (Life Technologies) according to the manufacturer’s instructions. qPCR was performed using a FastStart Universal SYBR Green kit (Roche) in an AP Biosystems RT-PCR machine. *act-3* was used as the control gene. qPCR data is presented as a percentage of wild-type expression levels. To analyze the qPCR data, copy number was calculated as 2^−Ct^ (Ct = cycle threshold). Next, copies/copy act-3 was calculated by dividing the copy number for each gene by the copy number for the control gene *act-3*. Finally, to express the data as “percent of wild-type”, all of the data points were divided by the average wild-type copies/copy act-3 for each gene.

### 2.5 Lifespan

For lifespan assays, pre-fertile young adult worms were picked from maintenance plates to lifespan plates containing 50 µM 5-fluoro-2′-deoxyuridine (FUdR). This concentration of FUdR completely prevents progeny from developing to adulthood and does not affect wild-type lifespan, but can affect the longevity of specific strains (Van Raamsdonk and Hekimi, 2011). Throughout the lifespan, worms were checked every 2–3 days for survival by observation and gently prodding worms that are not moving voluntarily. Three biological replicates started on three separate days were performed.

### 2.6 RNA interference

RNA interference (RNAi) was performed on plates containing 3 mM IPTG and 50 μg/mL carbenicillin. For lifespan experiments, worms were initially grown on RNAi plates with no FUdR. Once worms reached adulthood, they were transferred to RNAi plates containing 50 µM FUdR.

### 2.7 Statistical analysis

For lifespan studies, genotypes or RNAi clones were coded so that the identity of the strain or clones was not known by the experimenter. Graphpad PRISM Version 9.4.1 was used to prepare graphs and perform statistical analyses. Statistical significance for survival plots was determined using the log-rank test. For bar graphs, statistical significance was determined using a one-way ANOVA with Dunnett’s multiple comparisons test, a two-way ANOVA with Šidák’s multiple comparisons test or an unpaired t-test. *p < 0.05, **p < 0.01, ***p < 0.001, ****p < 0.0001.

## 3 Results

### 3.1 *fmo-2* expression is increased in long-lived mitochondrial mutants

Previous work has demonstrated an increase in *fmo-2* expression in long-lived *vhl-1* mutants and under dietary restriction, and that in both cases *fmo-2* is required to extend longevity (Leiser et al., 2015). In addition, overexpression of *fmo-2* is sufficient to increase lifespan (Leiser et al., 2015). Given the role of *fmo-2* in lifespan extension in these different contexts, we sought to determine the extent to which *fmo-2* also plays a role in the extended longevity of the long-lived mitochondrial mutants: *clk-1, isp-1* and *nuo-6* (Lakowski and Hekimi, 1996; Feng et al., 2001; Yang and Hekimi, 2010).

As a first step, we examined the expression of *fmo-2* in the long-lived mitochondrial mutants. To do this, we utilized RNA sequencing data from a recent study in which we compared genes expression across a panel of nine long-lived mutants (Rudich et al., 2025). In addition to the three long-lived mitochondrial mutants, this panel contained worms with decreased insulin/IGF-1 signaling (*daf-2*) (Kenyon et al., 1993), worms with dietary restriction (*eat-*2) (Lakowski and Hekimi, 1998), worms with decreased translation (*ife-*2) (Syntichaki et al., 2007), worms with decreased chemosensation (*osm-*5) (Apfeld and Kenyon, 1999), worms with germline ablation (*glp-1*) (Hsin and Kenyon, 1999) and worms with elevated mitochondrial superoxide (*sod-2*) (Van Raamsdonk and Hekimi, 2009). Interestingly, we previously found that these nine long-lived mutants cluster into three distinct groups based on gene expression (Rudich et al., 2025). The long-lived mitochondrial mutants are part of longevity group 1, which also contains *daf-2, glp-1* and *sod-2* worms, while *eat-2* and *osm-5* worms make up longevity group 2.

**FIGURE 1:**
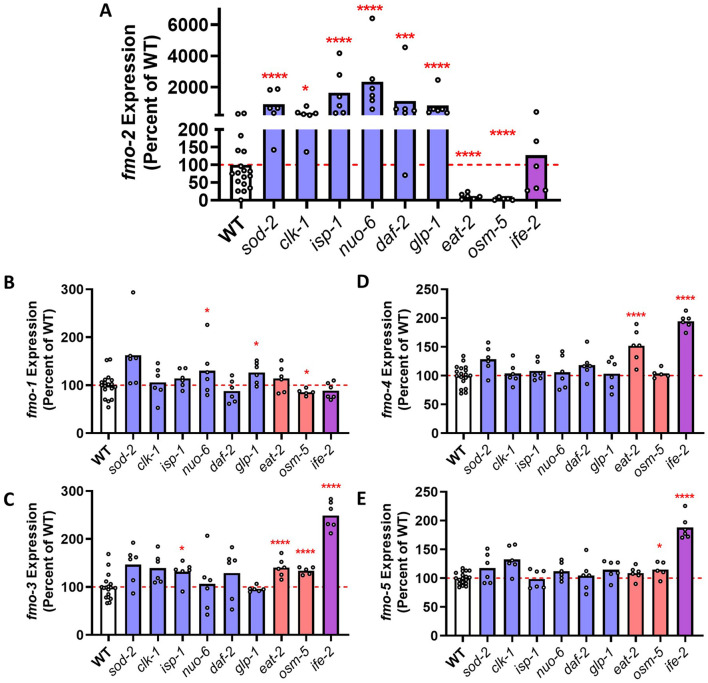
Expression of *fmo-2* mRNA is increased in long-lived mutants. **(A)** Long-lived mutants from longevity group 1 (*sod-2, clk-1, isp-1, nuo-6, daf-2, glp-1*) exhibit a significant upregulation of *fmo-2*, while long-lived mutants from longevity group 2 (*eat-2, osm-5*) show significantly decreased expression of *fmo-2. fmo-2* expression is unchanged in the group 3 longevity mutant *ife-2.* In contrast, expression of *fmo-1* **(B)** *, fmo-3* **(C)** *, fmo-4* **(D)** and *fmo-5* **(E)** are largely equivalent to wild-type in group 1 longevity mutants. *ife-2* mutants exhibit upregulation of *fmo-3, fmo-4* and *fmo-5.* The gene expression data used for this Figure is available on NCBI GEO: GSE179825, GSE93724 and GSE110984. The data can be accessed and analyzed using an online tool that we developed here: https://vanraamsdonk.shinyapps.io/mutant_comparison_viewer/.

In examining the expression of *fmo-2* across these nine long-lived mutants, we found that *fmo-2* mRNA is significantly increased in all six of the group 1 longevity mutants (*sod-2, clk-1, isp-1, nuo-6, daf-2, glp-1*), which includes the long-lived mitochondrial mutants (Figure 1A). The observation of increased *fmo-2* expression in *glp-1* worms is consistent with a previous study showing an increase in *glp-1* worms using an *fmo-2p::GFP* reporter strain (Goh et al., 2018). In contrast, *fmo-2* expression is significantly decreased in the group 2 longevity mutants *eat-2* and *osm-5* and unaffected in the group 3 *ife-2* mutants (Figure 1A)*.* These findings suggest that *fmo-2* does not contribute to the extended longevity of *eat-2, osm-5* or *ife-2* mutants.

We also examined the expression of the other four *fmo* genes in the panel of long-lived mutants. In contrast to *fmo-2,* the expression of the four other *fmo* genes was largely unchanged in the group 1 longevity mutants (Figures 1B–D). There was a small but significant upregulation of *fmo-3, fmo-4* and *fmo-5* in *ife-2* mutants but the magnitude of this increase was smaller than the upregulation of *fmo-2* in the group 1 longevity mutants. Combined, this indicates that the upregulation of *fmo* genes in the group 1 longevity mutants is specific for *fmo-2.*

### 3.2 *fmo-2* is required for lifespan extension in long-lived mitochondrial mutants

**FIGURE 2:**
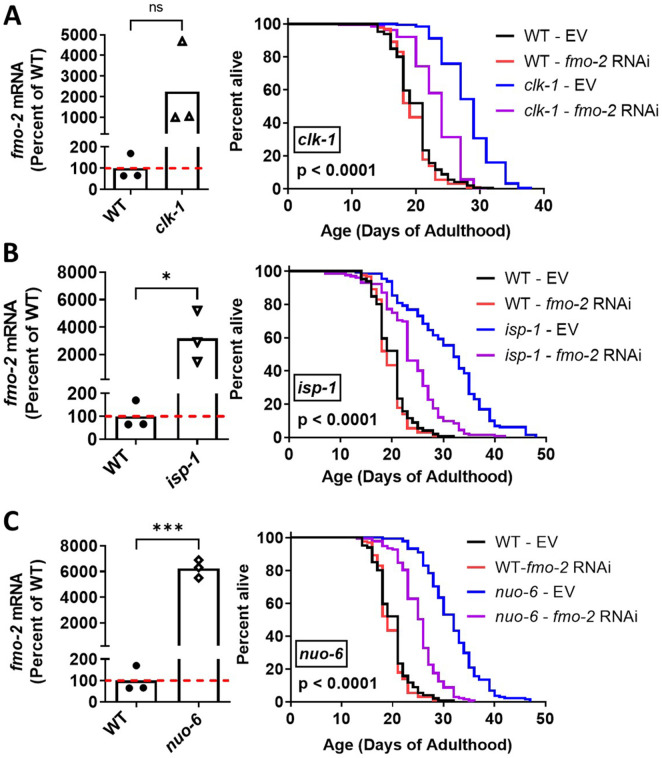
*fmo-2* is required for extended longevity in long-lived mitochondrial mutants. Quantification of *fmo-2* mRNA levels in day 1 pre-fertile young adult worms reveals that *fmo-2* mRNA levels are significantly increased in *clk-1* **(A)**, *isp-1* **(B)** and *nuo-6* **(C)** worms. Decreasing *fmo-2* levels through treatment with *fmo-2* RNAi significantly decreased the lifespan of *clk-1* **(A)**, *isp-1* **(B)** and *nuo-6* **(C)** worms despite having no effect on wild-type longevity. This suggests that the upregulation of *fmo-2* in these long-lived mutants is required for their extended longevity. Statistical significance was assessed using an unpaired t-test for *fmo-2* mRNA levels or a log-rank test for survival plots. For survival plots, p-value denotes the statistical significance between the blue and purple lines. Data for the effect of *fmo-2* RNAi on *nuo-6* lifespan is from Wu et al., 2018 *BMC Biology.* Raw lifespan data is provided in Supplementary Table S1.

Having shown that *fmo-2* expression is significantly increased in the long-lived mitochondrial mutants, we next sought to determine if this increase in *fmo-2* might be contributing to their longevity. We also sought to confirm the increase in *fmo-2* expression using quantitative RT-PCR (qPCR). Similar to the RNA sequencing results, we found that *fmo-2* expression is significantly increased in *clk-1, isp-1* and *nuo-6* worms using qPCR (Figures 2A–C). While *fmo-2* RNAi had no effect on the lifespan of wild-type worms, knocking down *fmo-2* significantly decreased the lifespan of all three mitochondrial mutants *clk-1, isp-1* and *nuo-6* (Figures 2A–C). In each case, the lifespan following *fmo-2* RNAi was not fully reduced to wild-type lifespan. This suggests that while *fmo-2* is required for the full lifespan extension in the long-lived mitochondrial mutants, other factors are also contributing to their longevity. Alternatively, since *fmo-2* RNAi does not result in the complete absence of *fmo-2,* it is possible that the residual *fmo-2* expression allows the mitochondrial mutants to still live longer than wild-type worms when *fmo-2* is being knocked down.

To provide additional support for the role of *fmo-2* in the longevity of the long-lived mitochondrial mutants, we crossed the mitochondrial mutants to an *fmo-2* deletion mutant (ok2147) and measured lifespan. The ok2147 mutation is a 1,070 bp deletion with a 2 bp insertion that affects exons 4 and 5 of 5 total exons. Consistent with the results from the *fmo-2* RNAi experiments, we found that deletion of *fmo-2* significantly decreased the lifespan of *clk-1* and *nuo-6* mutants (Supplementary Figure S1). Note that we were not able to generate *isp-1;fmo-2* double mutants as both genes are in close proximity on the same chromosome. Combined, the *fmo-2* RNAi and *fmo-2* deletion results demonstrate that *fmo-2* is required for the long lifespan of the long-lived mitochondrial mutants.

### 3.3 Transcription factors that activate *fmo-2* are required for longevity of long-lived mitochondrial mutants

Previous work has shown that the HLH-30 transcription factor is required for both the upregulation of *fmo-2* and lifespan extension resulting from HIF-1 activation, dietary restriction or *tald-1* knockdown (Leiser et al., 2015; Bennett et al., 2017). The p38 MAPK PMK-1 was also shown to be required for *fmo-2* upregulation in response to dietary restriction or *tald-1* RNAi, and proposed to act in parallel with HLH-30 (Bennett et al., 2017). Finally, it was found that MDT-15 and NHR-49 are required for the upregulation of *fmo-2* in response to organic peroxide, fasting or germline ablation (*glp-1* mutation), also in a HLH-30 independent manner (Goh et al., 2018).

**FIGURE 3:**
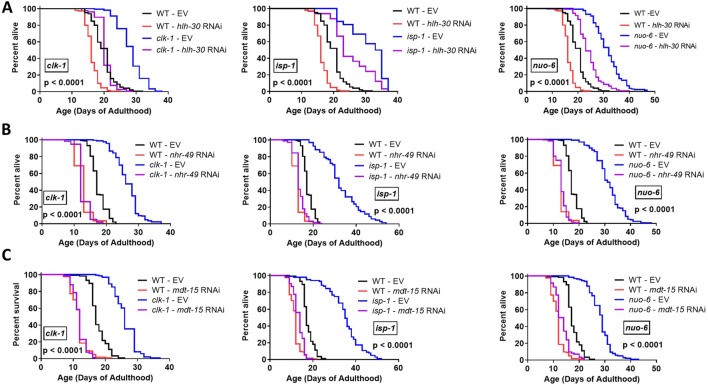
*hlh-30, nhr-49* and *mdt-15* are required for extended longevity in long-lived mitochondrial mutants and wild-type lifespan. Decreasing the expression of *hlh-30* **(A)**, *nhr-49* **(B)** or *mdt-15* **(C)** using RNAi decreased the lifespan of *clk-1, isp-1, nuo-6* and wild-type worms. The decrease in lifespan was partial for *hlh-30,* while the *clk-1, isp-1* and *nuo-6* mutations failed to increase lifespan when either *nhr-49* or *mdt-15* was knocked down. Statistical significance was assessed using a log-rank test. p-value denotes the statistical significance between the blue and purple lines. Raw lifespan data is provided in Supplementary Table S1.

Having shown that *fmo-2* is required for the longevity of the long-lived mitochondrial mutants, we sought to determine the extent to which genes upstream of *fmo-2* are also contributing to their long lifespans. To do this, we disrupted genes that were previously shown to be required for upregulation of *fmo-2* in the long-lived mitochondrial mutants and measured lifespan*.* We previously showed that disruption of genes involved in the p38-mediated innate immune signaling pathway (*nsy-1, sek-1, pmk-1*) are required for the long lifespan of *isp-1* and *nuo-6* worms (Campos et al., 2021). Knocking down *hlh-30* using RNAi significantly decreased the lifespan of *clk-1, isp-1* and *nuo-6* worms, but also reduced wild-type lifespan (Figure 3A). Similar to *fmo-2* knockdown, knockdown of *hlh-30* did not fully revert their lifespan back to wild-type treated with *hlh-30* RNAi. In contrast, we found that knocking down either *nhr-49* (Figure 3B) or *mdt-15* (Figure 3C) completely prevented lifespan extension resulting from mutations in *clk-1, isp-1* or *nuo-6*. As with *hlh-30, nhr-49* RNAi and *mdt-15* RNAi significantly decreased the lifespan of wild-type worms. While these lifespan results are consistent with *hlh-30, nhr-49* and *mdt-15* contributing to the longevity of the long-lived mitochondrial mutants, it is possible that disruption of these genes is having a generally toxic effect as disruption of these genes also decreases lifespan in wild-type worms. Nonetheless, these results demonstrate that pathways involved in the activation of *fmo-2* are also required for the longevity of the long-lived mitochondrial mutants. Further experiments would be needed to determine if these pathways are contributing to lifespan extension in these mutants or supporting it.

### 3.4 Pathways that mediate extended lifespan of *clk-1* mitochondrial mutants contribute to upregulation of *fmo-2*

In a previous study, we observed that deletion of *atfs-1* decreased both *fmo-2* levels and lifespan in *nuo-6* mutants (Wu et al., 2018), suggesting that pathways affecting lifespan in the mitochondrial mutants strains may also be affecting *fmo-2* levels. To test this idea, we decided to focus on *clk-1* mutants. In exploring the molecular mechanisms of lifespan extension in *clk-1* mutants, we have identified a number of genes that are required for the long life of *clk-1* mutants, some of which have been previously reported by us or others. As our results suggest a role for *fmo-2* in *clk-1* longevity, we wondered which genes that affect *clk-1* lifespan also affect the expression of *fmo-2.* To study this, we disrupted specific genes related to longevity (*daf-16, pmk-1, skn-1, ceh-23, aak-2, hif-1* and *elt-2*) using either a genetic mutation or RNAi and then used quantitative RT-PCR to measure the levels of *fmo-2* mRNA in wild-type and *clk-1* animals compared to empty vector (EV) control. We also measured lifespan in all four groups.

**FIGURE 4:**
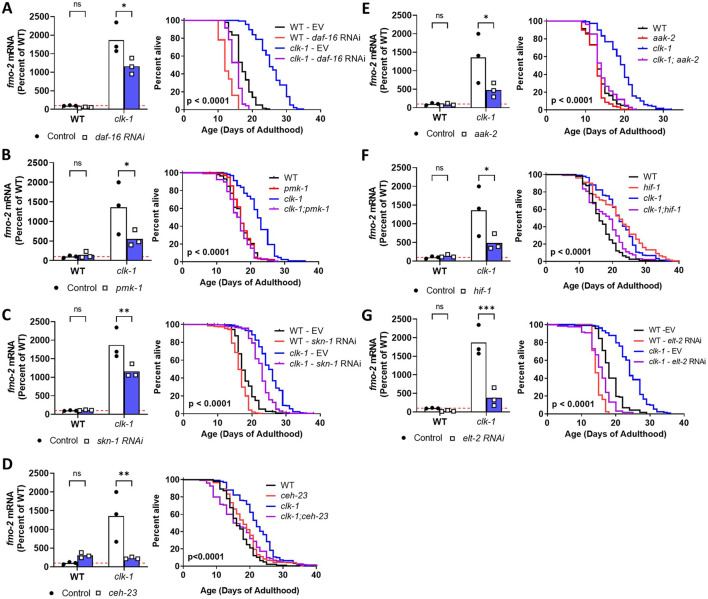
Pathways promoting longevity in long-lived *clk-1* mutants are specifically required for upregulation of *fmo-2* in *clk-1* worms. To better understand the relationship between *fmo-2* expression and lifespan in *clk-1* mutants, a number of lifespan-modulating pathways were disrupted in *clk-1* mutants using RNAi or genetic mutation before measuring lifespan and *fmo-2* expression. *fmo-2* expression was measured using qPCR in pre-fertile young adult worms. The genes that were disrupted included *daf-16* **(A)** *, pmk-1* **(B)**, *skn-1* **(C)**, *ceh-23* **(D)** *, aak-2* **(E)** *, hif-1* **(F)** and *elt-2* **(G)**. In every case, disruption of the longevity-related gene decreased *clk-1* lifespan and reduced *fmo-2* mRNA levels specifically in *clk-1* worms but not wild-type worms. Statistical significance was assessed using a two-way ANOVA with for Šidák’s multiple comparisons test for *fmo-2* mRNA levels or a log-rank test for survival plots. Lifespan data for DAF-16 is from Senchuk et al., 2018 *PLoS Genetics.* Lifespan data for PMK-1 is from Soo et al. (2023). *Aging Cell*. *p < 0.05, **p < 0.01, ***p < 0.001. Raw lifespan data is provided in Supplementary Table S1.

We previously showed that disruption of the FOXO transcription factor DAF-16 or the p38 homolog PMK-1 both decrease *clk-1* lifespan (Senchuk et al., 2018; Campos et al., 2021). Here, we extend these findings to show that *daf-16* RNAi (Figure 4A) or deletion of *pmk-1* (Figure 4B) both significantly decrease *fmo-2* levels in *clk-1* worms but not wild-type worms. The NRF2 homolog SKN-1 is involved in defending against oxidative stress and has been shown to be required for the long-lifespan of *daf-2* mutants (Tullet et al., 2008). We find that in *clk-1* mutants, RNAi targeting *skn-1* decreases *clk-1* lifespan and reduces the expression of *fmo-2* (Figure 4C). Previous work showed that the nuclear homeobox protein CEH-23 is required for lifespan extension in *clk-1, isp-1* and *nuo-6* mutants (Walter et al., 2011). Deletion of *ceh-23* completely reverts the elevated *fmo-2* expression in *clk-1* worms back to wild-type but does not significantly affect *fmo-2* levels in wild-type worms (Figure 4D). The AMP-activated protein kinase AAK-2 has been shown to be required for the long lifespan of *daf-2* worms (Apfeld et al., 2004) and worms exposed to a lifespan-extending dose of paraquat (Hwang et al., 2014). *aak-2* is also partially required for the longevity of *clk-1* and *isp-1* mutants (Curtis et al., 2006). In our hands, we find that deletion of *aak-2* reverts *clk-1* lifespan to wild-type and decreases the expression of *fmo-2* in *clk-1* worms (Figure 4E). Previous studies have shown that the hypoxia inducible factor HIF-1 is required for lifespan extension in *clk-1* and *isp-1* mutants (Lee et al., 2010). Here, we show that deletion of *hif-1* decreases both lifespan and *fmo-2* mRNA levels in *clk-1* worms (Figure 4F). The GATA transcription factor ELT-2 was shown to be required for wild-type lifespan and also the extended longevity of *daf-2, eat-2* and *isp-1* worms (Shore et al., 2012). RNAi targeting *elt-2* markedly decreases *clk-1* lifespan and reduces *fmo-2* expression specifically in *clk-1* mutants (Figure 4G). Combined, these results provide multiple examples of genetic pathways that are involved in both the longevity of *clk-1* worms and the expression of *fmo-2* in these mutants.

It is interesting to note that disruption of these longevity-related genes specifically reduced *fmo-2* levels in *clk-1* worms but did not significantly affect *fmo-2* levels in wild-type worms. In the case of *elt-2* and *daf-16* RNAi*,* we observed a trend towards decreased *fmo-2* levels, which failed to reach significance because of the large magnitude of *fmo-2* mRNA increase in *clk-1* worms. These results are consistent with the longevity-related genes (*daf-16, pmk-1, skn-1, ceh-23, aak-2, hif-1* and *elt-2*) being important for the upregulation of *fmo-2* in the long-lived mitochondrial mutants but being dispensable for basal expression levels.

## 4 Discussion

In this work, we show that *fmo-2* is required for the extended longevity of the long-lived mitochondrial mutants, *clk-1, isp-1* and *nuo-6*. We also identify additional upstream pathways that contribute to *fmo-2* upregulation and provide further support for the positive relationship between *fmo-2* expression levels and lifespan. At the same time, we identify two long-lived mutants, *eat-2* and *osm-5,* that provide an exception to this correlation, as they have extended longevity despite reduced expression of *fmo-2.*

Previous studies have shown that *fmo-2* is upregulated by several factors including hypoxia, *vhl-1* deletion, dietary restriction, *tald-1* RNAi, *cco-1* RNAi, exposure to exopolysaccharide-producing strains of lactobacilli, exposure to tert-butyl hydroperoxide (tBOOH) and germline ablation (*glp-1* mutation) (Leiser et al., 2015; Bennett et al., 2017; Goh et al., 2018). Here, we extend these findings to show that *fmo-2* is upregulated in a specific group of longevity mutants that includes *clk-1, isp-1, nuo-6, daf-2, glp-1* and *sod-2* mutants. We previously showed that this group of long-lived mutants exhibits activation of the DAF-16-mediated stress response and the mitochondrial unfolded protein response (Rudich et al., 2025).

Interestingly, long-lived mutants from a different longevity group, which includes *eat-2* and *osm-5* worms, exhibit a significant downregulation of *fmo-2.* This provides a clear example in which *fmo-2* expression levels and lifespan extension can be experimentally dissociated. It suggests that these long-lived mutants are utilizing a different strategy for living long that does not rely on *fmo-2.*

Previous work has shown that the HIF-1 hypoxia pathway is activated in the long-lived mitochondrial mutants *clk-1, isp-1* and *nuo-6,* and that *hif-1* is required for lifespan extension in all three mutants (Lee et al., 2010; Wu et al., 2018). Thus, it is plausible that *fmo-2* is activated in the mitochondrial mutants via the HIF-1 hypoxia pathway. This conclusion is supported by our observation that disruption of *hif-1* decreases *fmo-2* upregulation in *clk-1* worms. At the same time, it is likely that other pathways also contribute. In this work, we show that disruption of several signaling molecules that affect longevity are required for the upregulation of *fmo-2* in *clk-1* worms. In addition to *hif-1,* this includes *daf-16, pmk-1, skn-1, ceh-23, aak-2* and *elt-2.* Future epistasis experiments will be needed to sort out the extent to which these factors are working together or in parallel pathways to upregulate *fmo-2* expression.

Our work also shows that *hlh-30, nhr-49* and *mdt-15* are all required for the long-lifespan on *clk-1, isp-1* and *nuo-6* worms. As all of these genes have been implicated in the upregulation of *fmo-2*, it is plausible that their effect on lifespan results from an inhibition of *fmo-2* upregulation (Leiser et al., 2015; Goh et al., 2018). However, this interpretation is complicated by the observation that knocking down *hlh-30, nhr-49* or *mdt-15* decreases wild-type lifespan. Thus, the decrease in mitochondrial mutant lifespan could also just be due to a generally toxic effect of disrupting these important proteins. Previous research demonstrated that *hlh-30* RNAi also decreases lifespan in *daf-2, eat-2, glp-1, clk-1* and *rsks-1* long-lived mutants (Lapierre et al., 2013). In that paper, they observed enhanced levels of HLH-30 in the nuclei of intestinal cells of *daf-2, eat-2, clk-1* and *rsks-1* mutants, which is consistent with activation. In previous work, disruption of *nhr-49* was found to decrease the lifespan of *glp-1* worms, *isp-1* worms, *eat-2* worms, and worms treated with *cyc-1* RNAi, but have little or no effect on *daf-2* ilfespan (Khan et al., 2013; Chamoli et al., 2014; Ratnappan et al., 2014). Finally, knockdown of *mdt-15* using RNAi was found to shorten *daf-2* lifespan (Goh et al., 2013).

Importantly, our data not only shows that *fmo-2* is upregulated in the long-lived mitochondrial mutants but also that *fmo-2* and factors involved in *fmo-2* upregulation are required for their extended longevity. In contrast to *isp-1* point mutants, which were shown here to require *fmo-2* for their longevity, *fmo-2* is not required for *isp-1* RNAi to extend longevity (Leiser et al., 2015). While on the surface these results are contradictory, it was previously shown that the mechanisms of lifespan extension in *isp-1* mutants and *isp-1* RNAi are different (Yang and Hekimi, 2010). This difference in *fmo-2-*dependency provides additional support for this conclusion.

Overall, this work highlights the role of *fmo-2* in determining longevity in the long-lived mitochondrial mutants. It also advances our understanding of the molecular mechanisms that modulate the expression of *fmo-2*. Based on these results, it appears that a mild impairment of mitochondrial function leads to the activation of a number of pathways that increase *fmo-2* expression and promote longevity. In future studies, it will be important to determine the extent to which these pathways act together or in parallel in promoting longevity and *fmo-2* activation.

## Data Availability

The original contributions presented in the study are included in the article/Supplementary Material, further inquiries can be directed to the corresponding author.
