# Supplementary material for: Mild mitochondrial impairment activates overlapping longevity pathways converging on the flavin-containing monooxygenase FMO-2
Source: Front Aging. 2026 Mar 25;7:1808540. doi: 10.3389/fragi.2026.1808540 (PMC13058474; doi:10.3389/fragi.2026.1808540)
Supplement: Supplementary file 2 [file Image1.pdf]

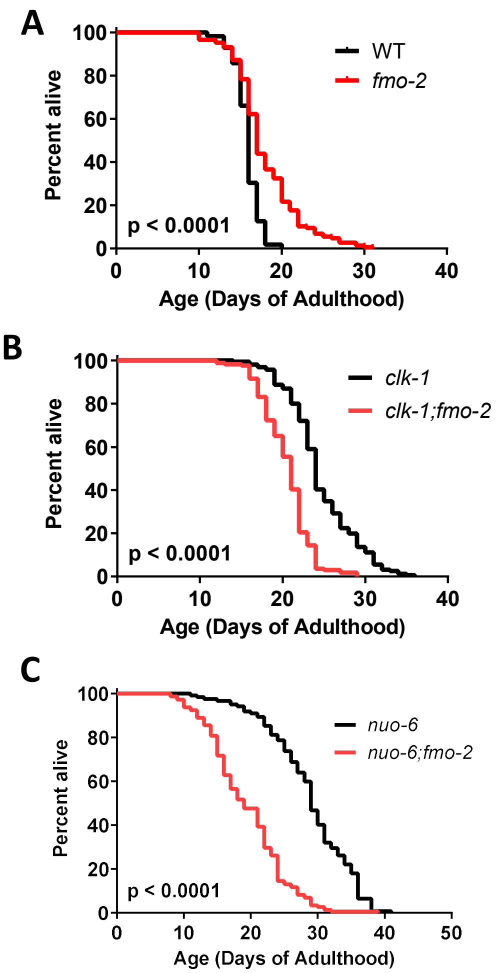

Figure S1. Deletion of *fmo-2* decreases the lifespan of long-lived mitochondrial mutants. While the *fmo-2* mutants exhibit increased lifespan in a wild-type background (**A**), deletion of *fmo-2* significantly reduces longevity in *clk-1* (**B**) and *nuo-6* (**C**) mutants. Statistical significance was assessed with a log-rank test.
